# Reassortment of Human Rotavirus Gene Segments into G11 Rotavirus Strains

**DOI:** 10.3201/eid1604.091591

**Published:** 2010-04

**Authors:** Jelle Matthijnssens, Mustafizur Rahman, Max Ciarlet, Mark Zeller, Elisabeth Heylen, Toyoko Nakagomi, Ryuichi Uchida, Zahid Hassan, Tasnim Azim, Osamu Nakagomi, Marc Van Ranst

**Affiliations:** Rega Institute for Medical Research, Leuven, Belgium (J. Matthijnssens, M. Zeller, E. Heylen, M. Van Ranst); International Centre for Diarrhoeal Disease Research, Bangladesh, Dhaka, Bangladesh (M. Rahman, Z. Hassan, T. Azim); Merck and Company, Inc., North Wales, Pennsylvania, USA (M. Ciarlet); Nagasaki University, Nagasaki, Japan (T. Nakagomi, R. Uchida, O. Nakagomi); 1 Current affiliation: Osaka University, Osaka, Japan.

**Keywords:** Rotavirus, G11, reassortment, Wa genogroup, genome, viruses, research

## Abstract

G11 rotaviruses are believed to be of porcine origin. However, a limited number of G11 rotaviruses have been recently isolated from humans in combination with P[25], P[8], P[6], and P[4]. To investigate the evolutionary relationships of these strains, we analyzed the complete genomes of 2 human G11P[25] strains, 2 human G11P[8] strains, and 3 porcine reference strains. Most of the 11 gene segments of these 7 strains belonged to genotype 1 (Wa-like). However, phylogenetic clustering patterns suggested that an unknown G11P[25] strain with a new I12 VP6 genotype was transmitted to the human population, in which it acquired human genotype 1 gene segments through reassortment, resulting in a human G11P[8] rotavirus strain with an entire human Wa-genogroup backbone. This Wa-like backbone is believed to have caused the worldwide spread of human G9 and G12 rotaviruses. G11 human rotavirus strains should be monitored because they may also become major human pathogens.

## Abstract 2

These viruses may become human pathogens.

Group A rotaviruses are the most frequently detected viral cause of diarrhea in children worldwide and cause ≈600,000 deaths in children <5 years of age annually, mainly in developing countries (*1*). Rotaviruses have a genome composed of 11 segments of double-stranded RNA that encodes 6 structural (VP) and 5 or 6 nonstructural (NSP) proteins (*2*). The 2 outer capsid proteins VP7 and VP4 are the basis for a widely used dual classification system defining G-types and P-types, respectively. Currently 23 G-genotypes and 31 P-genotypes have been described, of which 12 of each type have been found in human rotavirus isolates (*3**–**9*). However, only a limited number of G/P-genotype combinations are found frequently in humans, such as G1P[8], G2P[4], G3P[8], G4P[8], and G9P[8], and, more recently, the G12 genotype in combination with P[8] or P[6] (*10*). It has been hypothesized that the G9 and G12 genotypes have been able to successfully infect, spread, and persist in humans because of reassortment events with human Wa-like rotavirus strains, which has resulted in the G9 and G12 rotaviruses combining with P[8] and the 9 remaining gene segments belonging to genotype 1 (*10*).

A nucleotide sequence–based classification and nomenclature system encompassing the 11 rotavirus genome segments has been introduced recently, and it defines genotypes for each of the 11 gene segments (*11*). This system has been useful in investigating reassortment events and interspecies transmission of rotaviruses and their interhost relationships (*5**,**12**–**20*). In this system, the VP1–VP3, VP6, and NSP1–NSP5 genotypes comprising Wa-like strains have been designated as genotype 1 (R1, C1, M1, I1, A1, N1, T1, E1, and H1, respectively), and DS-1 and AU-1-like strains have been designated as genotypes 2 and 3, respectively (*11*). A Rotavirus Classification Working Group (RCWG) was formed to maintain and update this system and to assign successive genotype numbers to newly discovered rotavirus genotypes (*21*).

G11 rotaviruses are believed to be circulating in pigs, albeit in low numbers. Only 2 G11P[7] porcine strains have been isolated, strain YM in Mexico in 1983 and strain A253 in Venezuela in 1989 (*22**,**23*). Each of these porcine strains was identified as a single isolate in large strain collections obtained during epidemiologic surveys. In subsequent years, no additional G11 strains were detected in the same or nearby pig farms. However, more than a decade after the detection of these 2 porcine G11 strains, several reports have described the isolation of G11 rotavirus strains from humans. Three G11 rotaviruses, Dhaka6, KTM368, and CRI 10795, have been found in combination with the rare human genotype P[25] in India (*24*), Bangladesh (*25*), and Nepal (*26*). Furthermore, G11 human rotaviruses have been found in combination with P[8] (Matlab36–02 and Matlab22–01), with P[6] in Ecuador (EC2184) (*27*) and Bangladesh (Dhaka13–06) (*28*) and with P[4] (CUK-1) in South Korea (*29*).

It was recently suggested that human rotavirus strains belonging to the Wa-like genogroup and porcine rotaviruses have a common origin (*11*). This report prompted us to investigate the level of genetic relatedness, and possibly evolutionary origins, between these unusual human G11 rotavirus strains and porcine rotavirus strains by complete genome analyses of 2 human G11P[25] strains (KTM368 and Dhaka6), 2 human G11P[8] strains (Matlab36–02 and Dhaka22–01), and 3 cell culture–adapted porcine strains YM (G11P[7]), Gottfried (G4P[6]), and OSU (G5P[7]).

## Methods

### Strain Collection

Strain KTM368 (G11P[25]) was isolated in Nepal in 2004 (*26*), and rotavirus strains Dhaka6 (G11P[25]), Dhaka22–01 (G11P[8]), and Matlab36–02 (G11P[8]) were isolated in Bangladesh in 2001, 2001, and 2002, respectively (*25*,*28*). Double-stranded RNA of tissue culture–adapted reference porcine strains YM (G11P[7]), isolated in Mexico in 1983 (*23*), and OSU (G5P[7]) and Gottfried (G4P[6]) isolated in the United States in 1976 (*30*), was used in our study.

### RNA Extraction and Reverse Transcription–PCR

Virus RNA was extracted by using a QIAamp Viral RNA Mini Kit (QIAGEN, Leusden, the Netherlands) according to the manufacturer’s instructions. Extracted RNA was denatured at 97°C for 5 min, and reverse transcription–PCR was performed by using the OneStep RT-PCR Kit (QIAGEN). Forward and reverse primers used for amplification of different gene segments were synthesized on the basis of alignments of known 5′ and 3′ sequences of respective gene segments found in GenBank (primers are available upon request from J.M.). PCRs were performed by using an initial reverse transcription step at 50°C for 30 min, followed by PCR activation at 95°C for 15 min, 40 cycles of amplification, and a final extension at 72°C for 10 min in a BiometraT3000 Thermocycler (Biometra, Westburg, the Netherlands). Cycle conditions for the amplification of VP1, VP2, VP3, and VP4 were 30 s at 94°C, 30 s at 50°C, and 6 min at 70°C. For other gene segments, conditions were 30 s at 94°C, 30 s at 45°C, and 3 min at 72°C.

### Nucleotide Sequencing

PCR products were purified with the MSB Spin PCRapace Kit (Invitek, Berlin, Germany) and sequenced by using the dideoxy-nucleotide chain termination method with the ABI PRISM BigDye Terminator Cycle Sequencing Reaction Kit (Applied Biosystems, Foster City, CA, USA) in an ABI PRISM 3100 automated sequencer (Applied Biosystems). Complete 5′ and 3′ terminal nucleotide sequences of the 11 gene segments were determined by using a modified rapid amplification of cDNA ends technique as described (*15*).

### Nucleotide and Protein Sequence Analysis

Chromatogram sequencing files were analyzed by using Chromas 2.3 (Technelysium, Helensvale, Queensland, Australia), and contigs were prepared by using SeqMan II (DNASTAR, Madison, WI, USA). Multiple sequence alignments were constructed in ClustalX 2 (*31*) and subsequently edited in MEGA4 (*32*).

### Phylogenetic Analysis

Phylogenetic and molecular evolutionary analyses were conducted by using MEGA4 (*32*). Genetic distances were calculated by using the Kimura-2 correction parameter at the nucleotide level, and phylogenetic trees were constructed by using the neighbor-joining method with 500 bootstrap replicates.

### Assignment of Newly Identified Genotypes

Genotypes of each of the 11 genome segments for all the rotavirus strains under investigation were determined according to the genotyping recommendations of the RCWG by using the RotaC rotavirus genotyping tool (*21**,**33*). Because the sequence of the VP6 gene segment of strain KTM368 did not belong to any of the established VP6 I genotypes, it was submitted to the RCWG for appropriate genotype assignment. GenBank accession numbers for each of the gene segments of strains Dhaka6, KTM368, Dhaka22–01, Matlab36–02, YM, OSU, and Gottfried are shown in Technical Appendix 1.

## Results

Complete genome sequences of 3 human rotavirus strains (Dhaka6, KTM368, and Matlab36–02) were determined. For strain Dhaka22–01, only short nucleotide sequences ranging from 199 to 962 nt per segment could be determined because of insufficient sample. For porcine rotavirus strains YM, OSU, and Gottfried, variable amounts of gene sequences have been reported. We sequenced the remaining porcine rotavirus gene segments (Technical Appendix 1).

### Genotyping

According to guidelines of the RCWG, all gene segments belonged to established genotypes, except for the VP6 gene segment of KTM368 (*21*). This sequence was submitted to the RCWG, accepted as a new VP6 genotype, and designated I12. Complete genotype assignments of the 6 strains fully sequenced and reference strain Wa are shown in Table 1. All 3 human G11 strains have gene segments belonging to genotype 1 for the gene segments encoding VP1–VP3 and NSP1–NSP5. The VP4-encoding gene segment of human strains KTM368 and Dhaka6 had the unusual P[25] genotype, and human strains Matlab36–02 and Dhaka22–01 had the typical human P[8] genotype. With regard to VP6-encoding gene segments, only the human strain KTM368 from Nepal had the I12 genotype, and other human rotavirus strains isolated in Bangladesh had the typical human Wa-like VP6 genotype I1. The 3 porcine rotavirus strains YM, Gottfried, and OSU all had genotype 1 gene segments for VP1–VP3 and NSP2–NSP5 and the expected different G (G11, G4, and G5) and P (P[7] and P[6]) genotypes. The VP6 and NSP1 genotypes were either I1 or I5 and A1 or A8, respectively (Table 1).

**Table 1:** Genomic constellations of 3 human rotavirus strains, 3 porcine strains, and human reference strain Wa*

Strain	VP7	VP4	VP6	VP1	VP2	VP3	NSP1	NSP2	NSP3	NSP4	NSP5
Hu/Wa	G1	P[8]	I1	R1	C1	M1	A1	N1	T1	E1	H1
Hu/KTM368	G11	P[25]	I12	R1	C1	M1	A1	N1	T1	E1	H1
Hu/Dhaka6	G11	P[25]	I1	R1	C1	M1	A1	N1	T1	E1	H1
Hu/Matlab36–02	G11	P[8]	I1	R1	C1	M1	A1	N1	T1	E1	H1
Po/YM	G11	P[7]	I5	R1	C1	M1	A8	N1	T1	E1	H1
Po/Gottfried	G4	P[6]	I1	R1	C1	M1	A8	N1	T1	E1	H1
Po/OSU	G5	P[7]	I5	R1	C1	M1	A1	N1	T1	E1	H1

*VP, structural protein; NSP, nonstructural protein; Hu, human; Po, porcine. Wa genogroup genotypes are indicated in green, G11 and the rare P[25] and I12 genotypes are indicated in orange, and typical porcine genotypes are indicated in blue.

For several gene segments of the partially sequenced strains Dhaka22–01, insufficient sequence data were available for a definitive classification according to the guidelines of the RCWG (*21*). However, when available sequence data of strain Dhaka22–01 were compared pairwise with those of the other human G11 strains, sequences of VP7, VP6, and VP4 of strain Dhaka22–01 were nearly identical with those of the strain Matlab36–02 (99.8%, 99.0% and 98.4% identity at the nucleotide level, respectively) (Technical Appendix 1). In addition, for the VP1–VP2 and NSP1–NSP5 gene segments of strain Dhaka22–01, high identities (range 97.9%–100%) at the nucleotide level were found between strain Dhaka22–01 and the other human Wa-like G11 strains. For the VP3 gene segment, identities between strain Dhaka22–01 and strains KTM368, Dhaka6, and Matlab36–02 were low (range 86.3%–86.7%), and the VP3 gene segment of strain Dhaka22–01 was closely related to strains in the human M1 subcluster (99.6% identity with strain Dhaka12–03). These findings suggest a typical human Wa-like origin for Dhaka22-01 (Technical Appendix 1).

### Phylogenetic and Pairwise Identity Analyses

To study the relationships between human G11 and porcine rotavirus strains in greater detail, we constructed phylogenetic trees by using entire open reading frame nucleotide sequences for the 11 gene segments (Technical Appendix 2). For VP7, strains KTM368, Dhaka6, and Matlab36–02 cluster closely within the G11 genotype, together with the G11P[8] strain CUK1 from South Korea; the porcine G11 rotavirus strains YM and A253 are more distantly related (Technical Appendix 2). For VP6, strain KTM368 (genotype I12) was only distantly related to strains belonging to genotype I1. Human strains Dhaka6 and Matlab36–02 cluster in a large I1 subcluster, which contains mainly human and a few porcine strains isolated in Bangladesh, Belgium, the United States, Thailand, India, Australia, and Japan (Technical Appendix 2). For VP4, strains KTM368 and Dhaka6 cluster closely in the rare P[25] genotype, whereas strain Matlab36–02 is closely related to recently isolated P[8] human strains from Bangladesh, Belgium, South Korea, and the Democratic Republic of the Congo (Technical Appendix 2).

In the phylogenetic trees of the remaining 8 gene segments (VP1–VP3 and NSP1–NSP5), at least 1 major human monophyletic subcluster could be distinguished within genotype 1. We also observed 1 major porcine genotype 1 subcluster, a finding that is consistent with the assumption that Wa-like human rotavirus strains and porcine rotaviruses have a common ancestor (Technical Appendix 2) (*11*). Several gene segments of human G11 strains cluster closely in human genotype 1 subcluster, but a few of them did not cluster closely with any known human or porcine rotavirus strains and formed a distinct (nonhuman, nonporcine) branch or subcluster within genotype 1.

For the VP2 and NSP1 gene segments, human strains KTM368, Dhaka6, and Matlab36–02 clustered closely within the genotype 1 (C1 and A1, respectively) human subcluster (Technical Appendix 2). Regarding the VP1, NSP2, NSP3, NSP4, and NSP5 gene segments, human rotavirus strains Dhaka6 and Matlab36–02 are localized within the human subcluster, whereas strain KTM368 formed a distinct branch inside genotype 1 and did not cluster with any known human or porcine rotavirus strain (Technical Appendix 2). For VP3, all 3 human G11 strains (KTM368, Dhaka6, and Matlab36–02) belonged to a distinct subcluster inside the VP3 M1 genotype (Technical Appendix 2).

### Acquisition of Human Rotavirus Genes

The distinct genotype constellations (Table 2) differed in their degree of relatedness to typical human Wa genogroup rotavirus strains. With the exception of the G11 genotype, human strain Dhaka22–01 is nearly indistinguishable from other locally or more distantly circulating human Wa-genogroup rotavirus strains, such as strains Dhaka16–03 (G1P[8]) and Dhaka12–03 (G12P[6]) from Bangladesh or strain B4633–03 (G12P[8]) from Belgium, all isolated in 2003 (Technical Appendix 1) (*19*). Human strain Matlab36–02 is closely related to strain Dhaka22–01, except for the VP3 gene (Technical Appendix 2), for which human strains KTM368, Dhaka6, and Matlab36–02 form a distinct subcluster inside the M1 genotype. This finding suggests a recent reassortment event. Strain Dhaka6 is closely related to strain Matlab36–02 and only differs in the VP4 genotype (P[25] versus P[8]), respectively (Technical Appendix 2). This finding also suggests a recent reassortment event. Strain KTM368 from Nepal has VP2 and NSP1 gene segments that belong to the typical human subcluster within the C1 and A1 genotypes, respectively. All other gene segments belong to non–Wa-like genotypes (VP7: G11, VP4: P[25], and VP6: I12) or to a distinct subcluster or branch inside genotype 1 with an unknown origin.

**Table 2:** Subcluster-based genomic constellations of G11 human rotavirus strains, 3 porcine strains, and human reference strain Wa*

Strain	VP7	VP4	VP6	VP1	VP2	VP3	NSP1	NSP2	NSP3	NSP4	NSP5
Hu/Wa	G1	P[8]	I1	R1	C1	M1	A1	N1	T1	E1	H1
Hu/KTM368	G11	P[25]	I12	R1	C1	M1	A1	N1	T1	E1	H1
Hu/Dhaka6	G11	P[25]	I1	R1	C1	M1	A1	N1	T1	E1	H1
Hu/Matlab36–02	G11	P[8]	I1	R1	C1	M1	A1	N1	T1	E1	H1
Po/YM	G11	P[7]	I5	R1	C1	M1	A8	N1	T1	E1	H1
Po/Gottfried	G4	P[6]	I1	R1	C1	M1	A8	N1	T1	E1	H1
Po/OSU	G5	P[7]	I5	R1	C1	M1	A1	N1	T1	E1	H1

*VP, structural protein; NSP, nonstructural protein; Hu, human; Po, porcine. Genotypes belonging to the Wa genogroup indicated in green in Table 1 are further subdivided into subclusters shown in green, purple, and light blue, based on phylogenetic trees shown in Technical Appendix 2 (www.cdc.gov/EID/content/16/4/625-Techapp2.pdf), to distinguish additional patterns. G11 and the rare P[25] and I12 genotypes are indicated in orange, and typical porcine genotypes are indicated in dark blue.

There are 2 possible hypotheses for the observed acquisition of human rotavirus genes by G11 strains. The first hypothesis is that different human G11 virus strains described in this study may have originated from several unrelated interspecies transmission events of animal G11 strains to humans, followed by reassortment events that involved Wa-like human strains. The second hypothesis is that a gradual acquisition of human rotavirus genes occurred after 1 interspecies transmission event, followed by multiple successive reassortment events. The second hypothesis is that a currently unknown ancestral rotavirus, of probable porcine origin and having the G11-P[25]-I12 genotypes in a nonhuman Wa genogroup background, might have undergone multiple reassortment events with co-circulating human rotavirus strains, resulting in the different natural reassortant rotavirus strains described in this study. These reassortments resulted in a human G11P[8] rotavirus composed entirely of typical human genotype 1 (Wa-like) RNA segments.

## Discussion

G11 rotaviruses are considered porcine rotaviruses because they were first isolated from pigs in Venezuela and Mexico in the 1980s (*22**,**23**,**34*). Although G11 porcine rotaviruses have been detected infrequently on pig farms (*22**,**23**,**34*), these viruses have been recently detected in humans in several locations (India, Bangladesh, Nepal, South Korea, and Ecuador) (*24**–**29*). Our data show that multiple reassortment events have occurred between porcine or human G11 rotaviruses and co-circulating human Wa-like rotavirus strains (all human G11 strains were isolated during 2001–2006). In addition to G11 strains described in this study, another G11P[25] human rotavirus strain (CRI 10795) has been isolated in India, but only partial VP7, VP4, VP6, and NSP4 gene sequences of this strain are available (*24*). The CRI 10795 strain is yet another G11P[25] human rotavirus variant with a VP6 gene of the human I1 genotype and an NSP4 gene of the nonhuman subcluster of the E1 genotype. This finding suggests that additional reassortments have occurred between G11 and Wa-like strains.

Because the few human G11 strains investigated most likely represent only a small part of a complex set of events, our primary hypothesis of a linear stepwise acquisition of human rotavirus genes through successive reassortment events, which result in a human G11 rotavirus with an entire human Wa-like genomic background, may be oversimplified. An alternative hypothesis is that rare (porcine?) G11-P[25]-I12 progenitor strains are more widely spread and that genes of such viruses were introduced into the human population by interspecies transmissions, followed by multiple independent reassortment events with human rotavirus strains (G1P[8], G3P[8], G4P[8], G9P[8]). These reassortments could have resulted in different numbers of porcine gene segments being transferred to human Wa-like rotaviruses, as described for the G11 strains used in this study. Reassortment of individual or small numbers of porcine rotavirus genes into human rotaviruses, which are well adapted to propagation in the human host, can result in a virus with a genetic makeup that is optimal for replication in the human host and spread in the human population. However, close phylogenetic clustering of most genes of different G11 strains (Technical Appendix 2) suggests that these strains have a recent common ancestor and that gradual acquisition of human rotavirus genes is a plausible hypothesis.

The 2 hypotheses are not mutually exclusive, and a combination of both cannot be ruled out or proven at this time. In addition to G11P[25] and G11P[8] human rotaviruses, G11 rotaviruses in combination with P[4] and P[6] genotypes have been isolated in Bangladesh (*28*) and South Korea (*29*). However, additional sequence data are not available for these strains, which are suggestive for additional reassortment events with P[4] (DS-l like) and P[6] genotype rotaviruses. A recent report describes another human G11P[6] strain in Ecuador, which was found to be the likely result of reassortment between a typical porcine rotavirus and a human Wa-like rotavirus (*27*).

Early detection in ongoing surveillance programs and detailed analyses of G11 strains might provide unique insights into adaptation mechanisms of nonhuman rotaviruses to the human host through reassortment. G11 rotaviruses appear to be acquiring genotype 1 genome segments through multiple reassortment events (or Wa-like strains are acquiring genes of G11 rotavirus strains) in a short period. It will be useful to monitor whether new G11P[8] human rotavirus strains, which carry mainly human Wa-like genes, will be as successful as G9 and G12 rotaviruses in finding a niche in the human population, and whether the currently licensed rotavirus vaccines will afford protection against rotavirus disease caused by G11P[8] human rotavirus strains. Given that available rotavirus vaccines contain the virus P[8] component, it is more likely that they will also protect humans against G9, G11, and G12 strains with the VP4 genotype P[8].

## Supplementary Material

Technical Appendix GenBank Accession numbers, pairwise identities between partial nucleotide gene sequences.

Technical Appendix Phylogenetic trees based on full-length nucleotide sequences of rotavirus structural protein (VP1, VP2, VP3, VP4, VP6, and VP7) genes.
