# Supplementary material for: Reassortment of Human Rotavirus Gene Segments into G11 Rotavirus Strains
Source: Emerg Infect Dis. 2010 Apr;16(4):625–30. doi: 10.3201/eid1604.091591 (PMC3321964; doi:10.3201/eid1604.091591)
Supplement: Technical Appendix — GenBank Accession numbers, pairwise identities between partial nucleotide gene sequences. [file 09-1591-Techapp1.pdf]

Reassortment of G11 Rotavirus Strains in Humans

Table 1. GenBank accession numbers of rotavirus gene segments sequenced in the present study*

Gene	Dhaka6	Dhaka22–01	Matlab36–02	KTM368	YM	OSU	Gottfried
VP1	EF560705	GU199478	GU199503	GU199492	**X76486**	GU199514	**M32805**
VP2	GU199519	GU199479	GU199504	GU199493	GU199516	GU199515	GU199487
VP3	EF560706	GU199480	GU199505	GU199494	**AY300922**	**AY277921**	GU199488
VP4	GU199520	DQ482725	GU199506	GU199495	**M63231**	**X13190**	**M33516**
VP6	GU199521	GU199481	GU199507	GU199496	**X69487**	**AF317123**	**D00326**
VP7	*AY773003*	DQ482712	GU199508	GU199497	**M23194**	**X04613**	**X06386**
NSP1	GU199522	GU199482	GU199509	GU199498	**D38154**	**Z12107**	**U08431**
NSP2	EF560709	GU199483	GU199510	GU199499	GU199517	**X06722**	GU199489
NSP3	GU199523	GU199484	GU199511	GU199500	GU199518	**X81431**	**X81430**
NSP4	EF560711	GU199485	GU199512	GU199501	**X69485**	**D88831**	GU199490
NSP5	EF560712	GU199486	GU199513	GU199502	**X69486**	**X15519**	GU199491

*VP, structural protein; NSP, nonstructural protein. Numbers in **boldface** are accession numbers are from previous studies.

Table 2. Pairwise identities between partial nucleotide gene sequences of G11P[8] rotavirus strain Dhaka22–01 and other human G11 rotavirus strains (Matlab36–02, Dhaka6, and KTM368), human strains Dhaka16–03 (G1P[8]) and Dhaka12–03 (G12P[6]), and Belgian strain B4633–03 (G12P[8])*

Gene and strain	Strains						
VP1	Dhaka22_01	Matlab36–02	Dhaka6	KTM368	B4633–03	Dhaka12–03	Dhaka16–03
Dhaka22_01							
Matlab36–02	97.1						
Dhaka6	97.1	96.3					
KTM368	86.6	87.2	86.4				
B4633–03	97.9	97.6	98.7	86.1			
Dhaka12–03	100	97.1	97.1	86.6	97.9		
Dhaka16–03	97.1	96.9	97.9	85.9	99.2	97.1	
Wa	94.2	94	93.5	88.7	94.2	94.2	94
VP2							
Dhaka22_01							
Matlab36–02	93.5						
Dhaka6	93.5	100					
KTM368	93.3	99.4	99.4				
B4633–03	95.1	94.6	94.6	94.4			
Dhaka12–03	98.9	93.8	93.8	93.5	94.8		
Dhaka16–03	98.7	93.5	93.5	93.3	95.1	98.5	
Wa	91.4	92.7	92.7	92	91.6	91.2	91
VP3							

Dhaka22_01							
Matlab36–02	86.3						
Dhaka6	86.3	99.6					
KTM368	86.7	99.2	98.8				
B4633–03	99	85.7	85.7	86.1			
Dhaka12–03	99.6	86.3	86.3	86.7	99		
Dhaka16–03	94.8	86.7	86.7	87.5	93.8	94.4	
Wa	92.9	85.2	85.2	85.9	92.7	92.9	92.3

VP4							
Dhaka22_01							
Matlab36–02	98.4						
Dhaka6	58.5	58.4					
KTM368	58.4	58.3	99.5				
B4633–03	98	99.4	58.4	58.3			
Dhaka12–03	72.5	72.3	60.1	60	72.1		
Dhaka16–03	97.9	99.5	58.5	58.4	98.9	72.3	
Wa	91.5	91.6	58.8	58.6	91.5	72.1	91.2
VP6							

Dhaka22_01							
Matlab36–02	99						
Dhaka6	99.2	99.3					
KTM368	83.9	84.3	84.2				
B4633–03	97.5	97.1	97.2	84.2			
Dhaka12–03	98	98.2	98.3	84.2	97.1		
Dhaka16–03	98.3	98.2	98.3	83.9	97.3	99.4	
Wa	90.5	90.5	90.3	83.5	91.2	90.2	90.2
VP7							

Dhaka22_01							
Matlab36–02	99.8						
Dhaka6	99.5	99.7					
KTM368	99.7	99.5	99.8				
B4633–03	72.7	72.5	72.8	73			
Dhaka12–03	72.7	72.5	72.8	73	97.7		
Dhaka16–03	70.5	70.4	70.7	70.8	70.8	70.2	
Wa	70.2	70.4	70.7	70.5	70.4	70.2	91.1
NSP1							

Dhaka22_01							
Matlab36–02	98.3						

Dhaka6	97.9	99.2					
KTM368	99	98.6	98.2				
B4633–03	97.1	98	97.8	97.4			
Dhaka12–03	99.1	99	98.6	99.3	97.8		
Dhaka16–03	97.9	99.6	98.8	98.2	97.7	98.6	
Wa	85.5	85.7	85.5	85.3	85.3	85.7	86.1
NSP2							

Dhaka22_01							
Matlab36–02	99						
Dhaka6	98.8	99.6					
KTM368	88.1	88.1	88				
B4633–03	98.3	98.6	98.4	87.2			
Dhaka12–03	96.7	97.3	97.1	87.4	97.4		
Dhaka16–03	98.8	99.6	99.5	88	98.4	97.4	
Wa	93.6	93.5	93.1	87.4	93.6	92.8	93.3
NSP3							

Dhaka22_01							
Matlab36–02	97.6						
Dhaka6	97.7	99.3					
KTM368	92.5	92.5	92.9				
B4633–03	98.8	98	98.1	93.2			
Dhaka12–03	99.3	98	98.1	92.9	99.2		
Dhaka16–03	98	99.6	99.5	92.6	98.1	98.4	
Wa	95.6	95.1	95.2	92.8	96	96	95.2
NSP4							

Dhaka22_01							
Matlab36–02	100						
Dhaka6	99	99					
KTM368	87.8	87.8	87.8				
B4633–03	99	99	97.9	88.2			
Dhaka12–03	98.3	98.3	97.3	87.7	97.9		
Dhaka16–03	99.3	99.3	99.3	87.5	98.3	97.6	
Wa	93.7	93.7	93	89	93	92.8	93
NSP5							

Dhaka22_01							
Matlab36–02	99.5						
Dhaka6	98.6	98.1					
KTM368	94.5	94	95.6				
B4633–03	98.6	98.4	98.6	94.9			

Dhaka12–03	99.1	98.6	99.1	95.4	98.8		
Dhaka16–03	99.3	99.8	97.9	93.8	98.2	98.4	
Wa	92.8	92.3	93.1	92.4	92.4	93.3	92.3

*Values are percentages. VP, structural protein; NSP, nonstructural protein. The following nucleotide regions were used for comparisons: VP1: 26–224; VP2: 27–490; VP3: 303–797; VP4: 41–842; VP6: 42–755; VP7: 49–1010; NSP1: 19–737; NSP2: 41–770; NSP3: 35–771; NSP4: 62–732; NSP5: 38–645.
