# Supplementary material for: Reassortment of Human Rotavirus Gene Segments into G11 Rotavirus Strains
Source: Emerg Infect Dis. 2010 Apr;16(4):625–30. doi: 10.3201/eid1604.091591 (PMC3321964; doi:10.3201/eid1604.091591)
Supplement: Technical Appendix — Phylogenetic trees based on full-length nucleotide sequences of rotavirus structural protein (VP1, VP2, VP3, VP4, VP6, and VP7) genes. [file 09-1591-Techapp2.pdf]

Reassortment of G11 Rotavirus Strains in Humans

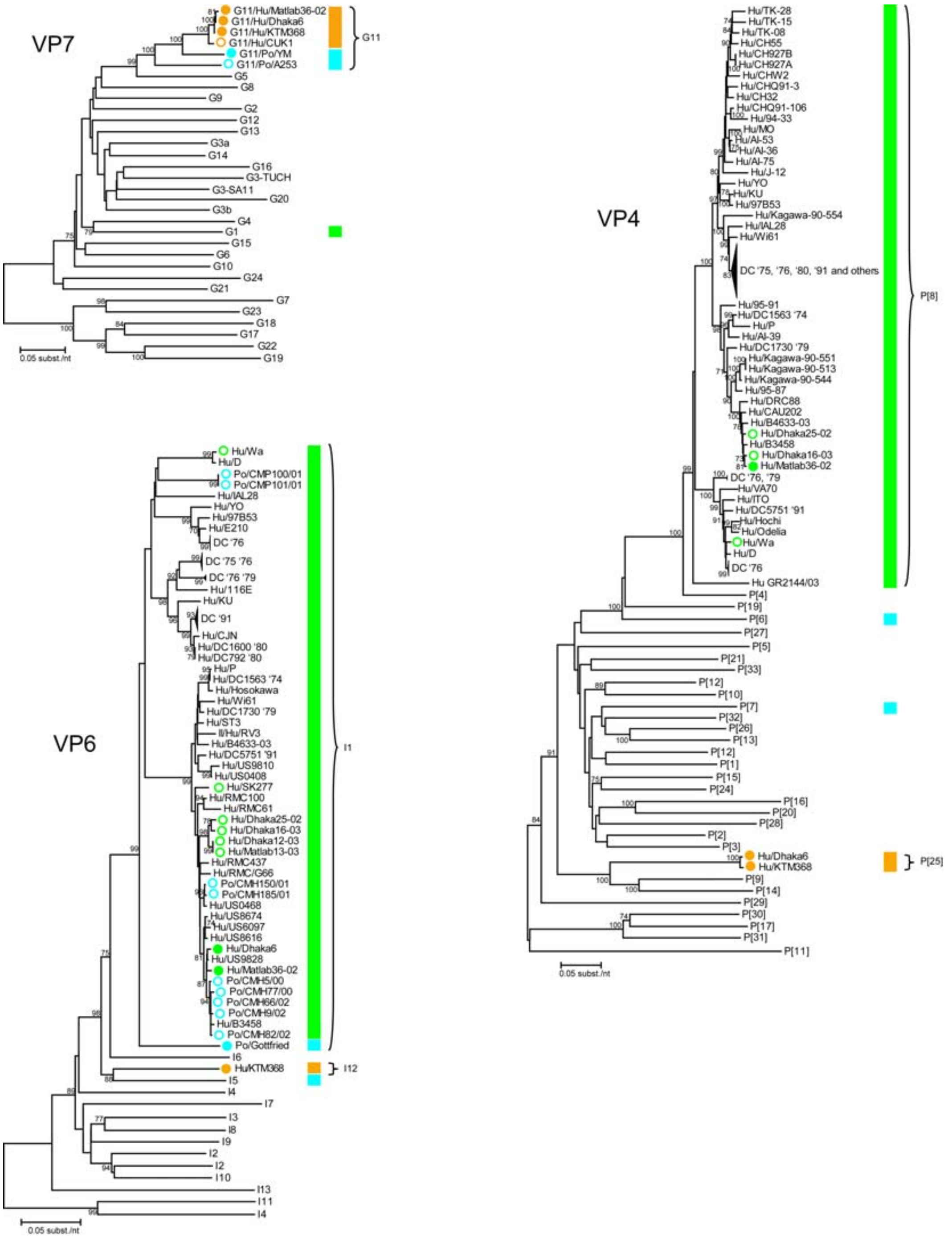

Figure 1. Phylogenetic trees based on full-length nucleotide sequences of rotavirus structural protein (VP4, VP6, and VP7) genes. Bootstrap values (500 replicates) >70 are shown. Only genotypes in which

the strains under investigation cluster are shown. Other genotypes are represented by genotype number. Species of origin/strain name is shown for each strain. Strains sequenced in this study are indicated by solid circles (green or orange for human strains and blue for porcine strains). Other porcine strains are indicated by open blue circles, and other human strains isolated in the same region as human G11 strains (and reference strain Wa) are indicated by open green circles. The open orange circle indicates novel human rotavirus strain CUK1. Green bars indicate major human Wa genogroup subclusters, blue bars indicate porcine subcluster(s), and orange bars indicate unusual human VP4, VP6, and VP7 genotypes P[25], I12, and G11, respectively. Hu, human; PO, porcine. subst/nt, substitutions per nucleotide.

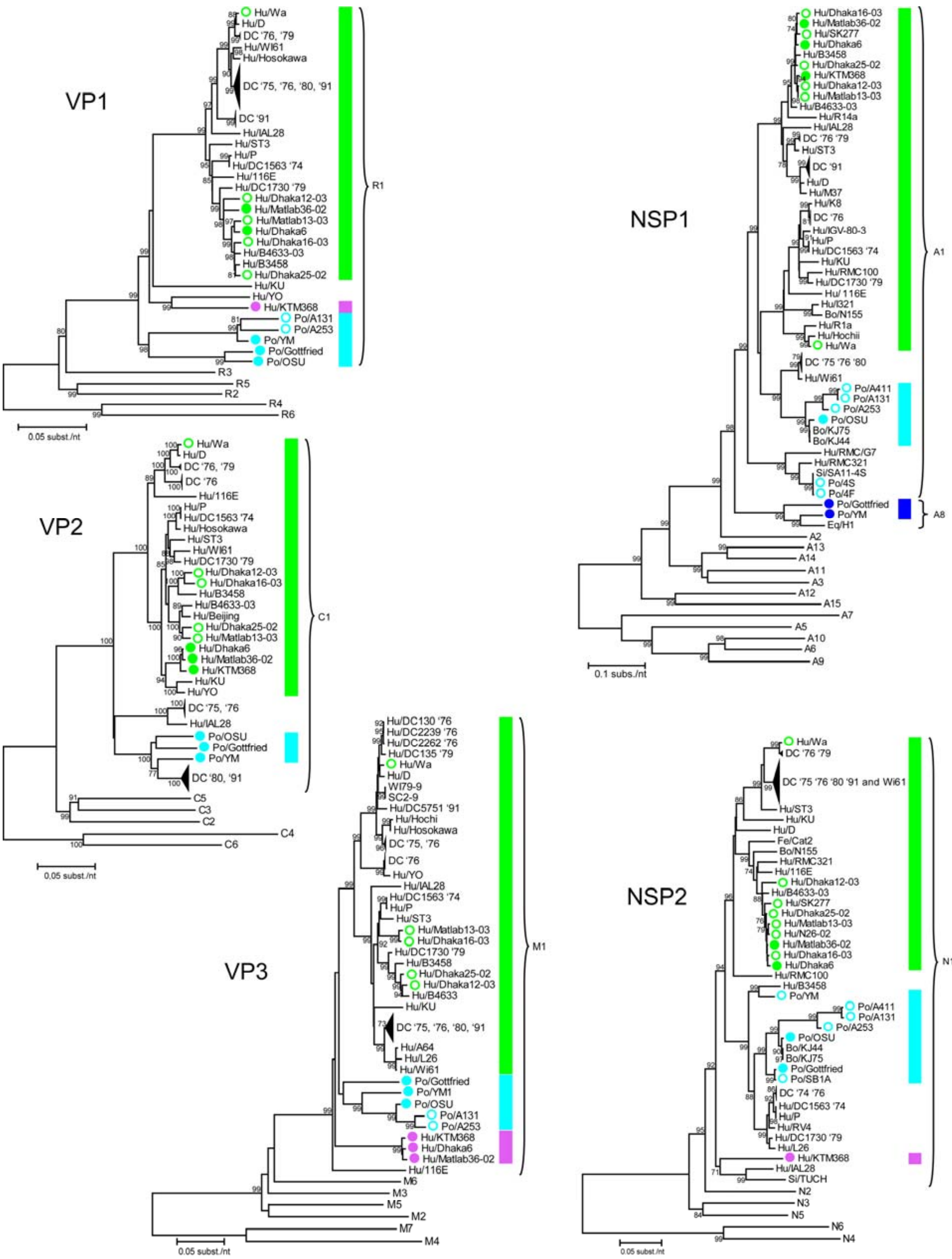

Figure 2. Phylogenetic trees based on full-length nucleotide sequences of rotavirus structural protein (VP1, VP2, and VP3) genes and nonstructural protein (NSP1 and NSP2) genes. Strains sequenced in

this study are indicated by solid circles (green or violet for human strains and blue or dark blue for porcine strains). Other porcine strains are indicated by open blue circles, and other human strains isolated in the same region as human G11 strains (and reference strain Wa) are indicated by open green circles. Green bars indicate major human Wa genogroup subclusters, light blue bars indicate porcine subclusters, dark blue bar indicates porcine rotaviruses with the A8 NSP1 genotype, and violet bars indicate unusual nonhuman, nonporcine Wa-like human G11 rotavirus genes. Hu, human; Po, porcine. subst/nt, substitutions per nucleotide.

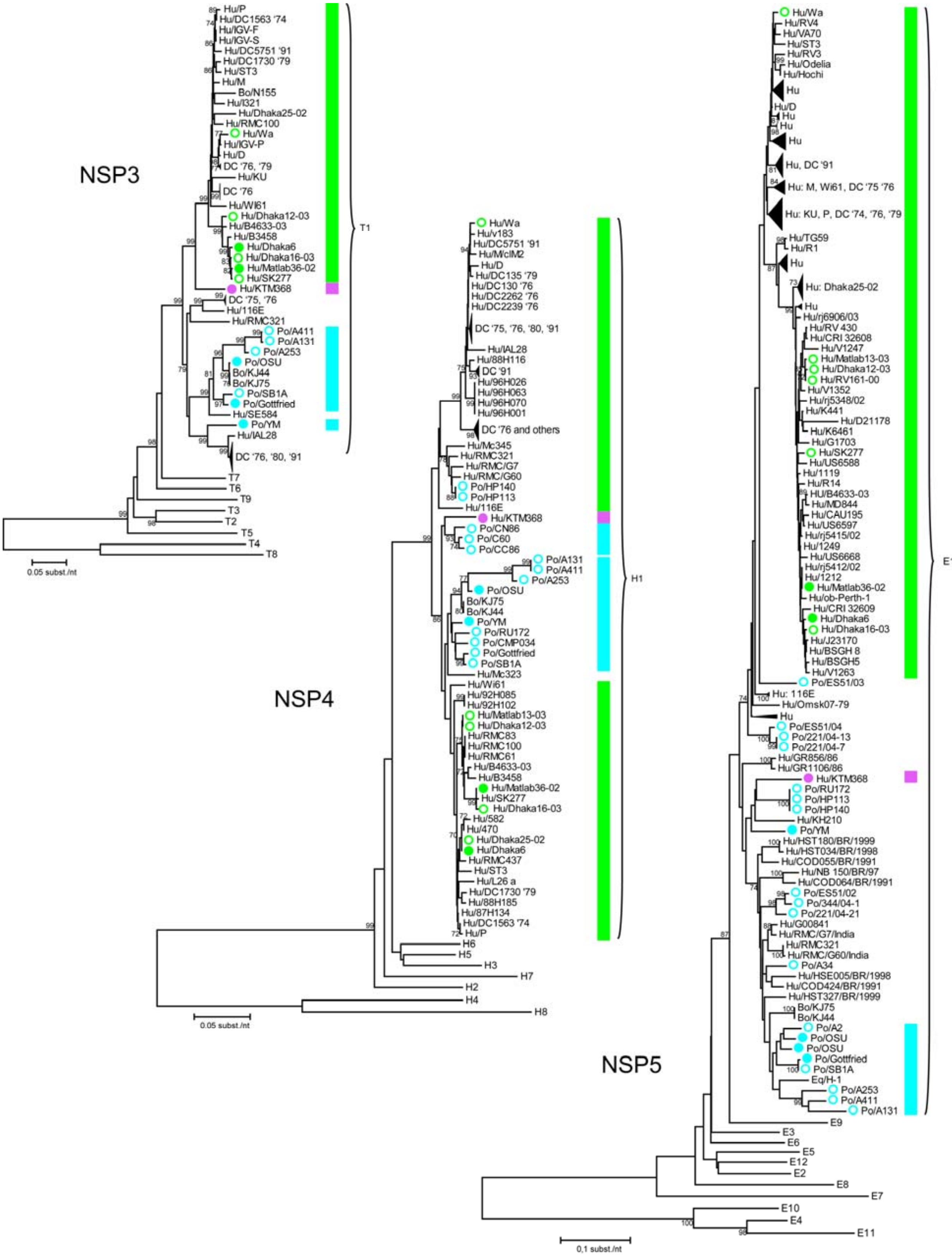

Figure 3. Phylogenetic trees based on full-length nucleotide sequences of rotavirus nonstructural protein (NSP3, NSP4, and NSP5) genes. Strains sequenced in this study are indicated by solid circles (green or

violet for human strains and blue for porcine strains). Other porcine strains are indicated by open blue circles, and other human strains isolated in the same region as human G11 strains (and reference strain Wa) are indicated by open green circles. Green bars indicate major human Wa genogroup subclusters, blue bars indicate porcine subclusters, and violet bars indicate unusual nonhuman, nonporcine Wa-like human G11 rotavirus genes. Although NSP4 and NSP5 genes of the KTM368 cluster more closely with porcine strains than with human strains, the genetic distance (branch length) is high enough so that they are not placed in the porcine subcluster. Hu, human; Po, porcine. subst/nt, substitutions per nucleotide.
